# Intra-Articular Analgesia and Steroid Reduce Pain Sensitivity in Knee OA Patients: An Interventional Cohort Study

**DOI:** 10.1155/2014/710490

**Published:** 2014-05-07

**Authors:** Tanja Schjødt Jørgensen, Thomas Graven-Nielsen, Karen Ellegaard, Bente Danneskiold-Samsøe, Henning Bliddal, Marius Henriksen

**Affiliations:** ^1^Clinical Motor Function Laboratory, The Parker Institute, Department of Rheumatology, Copenhagen University Hospital, Nordre Fasanvej 57, Bispebjerg and Frederiksberg, 2000 Frederiksberg, Denmark; ^2^Laboratory for Musculoskeletal Pain and Motor Control, Center for Sensory-Motor Interaction (SMI), Department of Health Science and Technology, Faculty of Medicine, Aalborg University, 9220 Aalborg, Denmark

## Abstract

*Objectives*. To assess the effects of intra-articular therapy on pain sensitivity in the knee and surrounding tissues in knee OA patients. *Methods*. Twenty-five knee OA patients with symptomatic knee OA were included in this interventional cohort study. Pressure pain thresholds (PPT) were recorded before, immediately after, and two weeks after ultrasound guided intra-articular injection of lidocaine combined with glucocorticosteroid. Computer-controlled and manual pressure algometers were used to assess PPT on the knee, vastus lateralis, tibialis anterior, and the extensor carpi radialis longus muscles (control site). *Results*. Significantly increased PPTs were found following intra-articular injection, at both the knee (*P* < 0.0001) and the surrounding muscles (*P* < 0.042). The treatment effects were sustained for two weeks, and at some points the effect was even greater at two weeks (*P* < 0.026). Albeit not statistically significant, a similar trend was observed at the control site. *Conclusions*. Intra-articular anesthesia, combined with glucocorticosteroid, reduced pain sensitivity in both the knee and surrounding muscles for at least two weeks.

## 1. Introduction

Osteoarthritis (OA) is a common chronic pain condition. There is growing consensus that knee OA involves a low-grade inflammation contributing to structural disease progression and generation and maintenance of pain [1–3]. Synovial inflammation acts as a trigger for several signs and symptoms of OA, including stiffness, effusion, and joint swelling [1], and has profound effects on the nociceptive system where cytokines seem to be a major player in the production of such effects [2]. Pain research has revealed that both the sensitisation of nociceptors located in deep tissue in the joint (peripheral sensitisation) and the sensitisation of spinal cord neurons with joint input (central sensitisation) are basic neuronal processes underlying pain and mechanical hyperalgesia in the inflamed joint [4, 5].

Recently, hyperalgesia and widespread pain were shown in knee OA patients, as both the knee and the surrounding sites were hyperalgesic to pressure pain stimulations compared with healthy controls [6]. Moreover, total knee replacement reduced the knee hyperalgesia and sensitisation phenomena, besides reducing the clinical pain intensity [7]. This indicates that intra-articular structures are involved in generation and maintenance of the pain sensitisation phenomena in knee OA.

Intra-articular glucocorticosteroid therapy is long established as having a moderate effect size unlike most OA therapies and widely used in clinical practice [8]. The mechanism of the therapeutic effect in knee OA is likely related to its potent anti-inflammatory effect [9–11], but to predict the analgesic response it is important to have a better understanding of the pain sensitisation phenomena in patients with knee OA [12] and the possible effects of glucocorticosteroid on this. Intra-articular glucocorticosteroid seems to be a good clinical model of analgesia to explore basic pain mechanisms in a clinical OA population.

The purpose of this study was to assess the effects of intra-articular analgesia and glucocorticosteroid injections on pain sensitivity and pain intensity in the knees and surrounding tissues of patients with knee OA. It was hypothesized that intra-articular analgesia and anti-inflammation reduce the pain sensitivity.

## 2. Patients and Methods

### 2.1. Patients

This was an interventional study conducted in Copenhagen, Denmark. Participants were recruited in March–December 2012 from the OA outpatients clinic of Copenhagen University Hospital at Frederiksberg, Copenhagen, Denmark. Eligible participants were adults aged 40 or over with a clinical diagnosis of tibiofemoral OA confirmed by radiography and an average maximum daily knee pain during the last week above 3 rated on a 0–10 numerical rating scale. Exclusion criteria included glucocorticosteroid injections in the previous 3 months, polyneuropathy, total hip and knee replacements, low back pain, and nerve root compression syndromes. Furthermore, the patients were requested not to take analgesic medication 24 h before tests. At inclusion, the most symptomatic knee was deemed the target knee for subsequent assessments and intra-articular treatment. The study was approved by the local ethics committee (j. number HC-2007-0053) and was conducted in accordance with the Declaration of Helsinki, and all subjects provided written informed consent.

### 2.2. Study Design Overview

Each patient was tested on 3 days over a period of 3 weeks. Two pretherapy baseline tests, separated by one week, were completed: baseline 1 (test 1) and baseline 2 (test 2). Immediately after the baseline 2, ultrasound guided intra-articular injection of lidocaine combined with glucocorticosteroid was administered (see below). The immediate effects of the intra-articular injection were assessed immediately after the injection (test 3). Two weeks later, all tests were repeated (test 4).

### 2.3. Intra-Articular Analgesia and Anti-Inflammatory Treatment

Intra-articular bolus injections of 10 mL lidocaine (10%) and 40 mg glucocorticosteroid (depomedrol) were administrated as a mixture with a lateral approach into the knee joint. The injection was ultrasound guided to ensure proper placement of the bolus injection in the suprapatellar bursa.

### 2.4. Outcomes

The primary outcome was pain sensitivity assessed as pressure pain thresholds (PPTs) acquired by computer-controlled and manual pressure algometers. Secondary outcomes included self-reported knee OA pain intensity.

### 2.5. Computer-Controlled Pressure Algometry

The computer-controlled pressure algometry is a standardised and objective method to gain important information on hyperalgesia. In the present study the custom built computer-controlled pressure algometer (Aalborg University, Denmark) was used for measuring computer-controlled PPTs at three sites: infrapatellar fat pad (2 cm distal to the inferior medial edge of patella), m. vastus lateralis (7 cm from the lateral upper rim of patella), and m. tibialis anterior (10 cm below the tibial tuberosity) as previously applied [13]. The computer-controlled pressure algometer applied the mechanical stimuli perpendicular to the skin surface [14]. A round aluminum probe with a padded contact surface of 1 cm^2^ was fixed to the tip of a piston. The pressure stimulation was feedback controlled via recordings of the actual force. The computer-controlled pressure stimulus, with an ascending pressure gradient of 60 kPa/s, was applied continuously until the subject reported the first sensation of pain and pressed a button. This recorded pressure intensity defined the baseline PPT. The PPT of each assessment site was recorded three times and used for further analysis.

### 2.6. Manual Pressure Algometry

To assess pain sensitivity at sites where computer-controlled pressure algometry was not feasible, a hand-held pressure algometer (Algometer Type II, Somedic AB, Sweden) was used to assess manual PPTs. Manual PPTs were assessed on 8 sites on the knee identified from bony landmarks as previously applied [13]: site 1: 2 cm distal to the inferior medial edge of patella; site 2: 2 cm distal to the inferior lateral edge of patella; site 3: 3 cm lateral to the midpoint on the lateral edge of patella; site 4: 2 cm proximal to the superior lateral edge of patella; site 5: 2 cm proximal to the superior edge of patella; site 6: 2 cm proximal to the superior medial edge of patella; site 7: 3 cm medial to the midpoint on the medial edge of patella; site 8: at centre of patella. The pressure was applied with a rate of approximately 30 kPa/s (visual feedback was provided), with a 1 cm^2^ probe. All participants were instructed to push a button when they felt that the pressure was just barely painful, defining the manual PPT. The PPT was measured twice on each assessment site and an interval of minimum 20 s was kept between each PPT assessment. The mean of all recordings for the 8 sites on the knee was calculated to assess the overall knee PPT.

Further, manual PPT was assessed on a control site on the contralateral arm at the extensor carpi radialis longus muscle 5 cm distal to lateral epicondyle of humerus as previously applied [6]. On this site PPT was also measured twice and averaged with an interval of minimum 20 s between each PPT assessment.

### 2.7. Current Knee OA Pain Intensity

At each visit the patients rated their current knee pain intensity on an electronic visual analogue scale (VAS) in which “0 cm” represented no pain and “10 cm” represented “maximal pain.”

### 2.8. Statistics

The data are presented as mean and 95% confidence intervals (95% CI). In the analysis of the PPTs and VAS scores for current knee pain a longitudinal data model was applied to assess multiple repeated-measures on the same subject using the MIXED procedure of the SAS system with random effect for subject (random intercept model). The analyses focused on the fixed effects analyses, analysing whether there were effects of time (4 levels: baseline 1 (test 1), baseline 2 (test 2), immediately after the injection (test 3), and two weeks after the injection (test 4)). Post hoc *t*-tests were used to explore the differences between assessment time points.

To explore if preinjection pain sensitivity was associated with self-reported treatment outcome on current knee pain, we assessed the correlation (Pearson's *r*) between manual PPT at test 2 and changes in current knee OA pain ratings from tests 2 to 3 and 2 to 4. Also, the correlation (Pearson's *r*) between changes in manual PPT and changes in current knee pain was assessed. We explored our data for normality and satisfaction of parametric statistics, and no transformations were required. All analyses were done using SAS software, and statistical significance was accepted at *P* < 0.05.

## 3. Results

**Figure 1:**
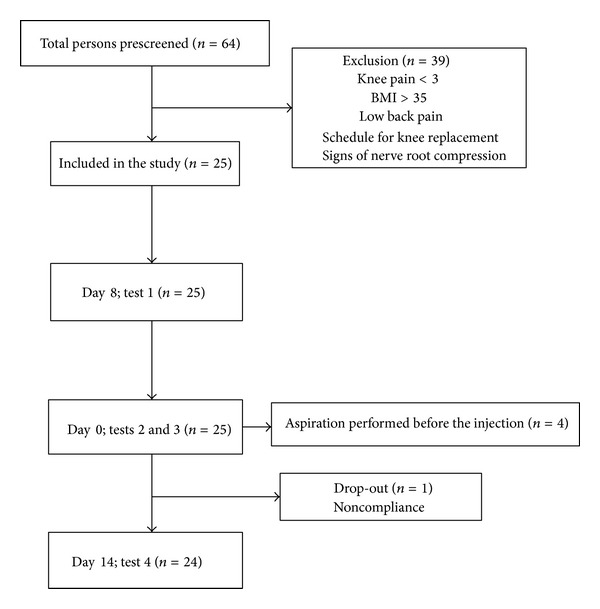
The progress and timeline of participants through the study.

A total of 64 patients were screened for the eligibility. Of those, 25 knee OA patients with symptomatic knee OA were included in this interventional cohort study (Figure 1). Four patients had excessive joint fluid aspirated before the intra-articular injection. Characteristics of the included patients are shown in Table 1. Twenty-four patients completed the study as one patient did not return for the final data collection (Figure 1).

### 3.1. Computer-Controlled Pressure Algometry

**Figure 2:**
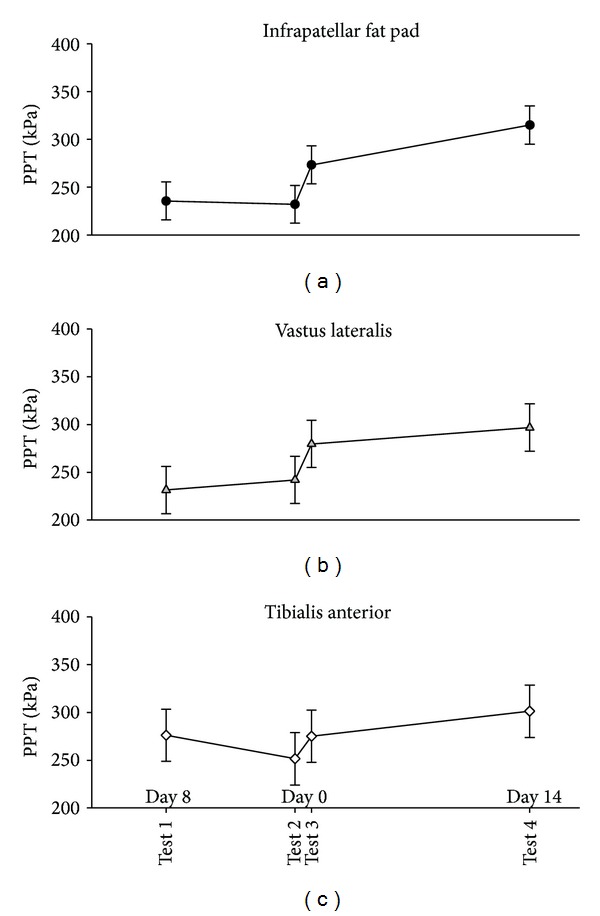
Mean computer-controlled PPTs (95% CI, *N* = 25 at tests 1, 2, and 3, *N* = 24 at test 4) on the infrapatellar fat pad (a), vastus lateralis (b), and tibialis anterior (c) before (baseline tests 1 and 2), immediately after (test 3), and two weeks after (test 4) the injection of lidocaine and glucocorticosteroid. PPTs were significantly increased at all three test sites compared with baseline 2 (test 2) (*P* > 0.042) immediately after (test 3) and further significantly increased two weeks after (test 4) the injection compared to immediately after the injection (test 3) on the infrapatellar fat pad (*P* = 0.0002) and tibialis anterior (*P* = 0.026), but not at vastus lateralis (*P* = 0.093).

In the computer-controlled PPTs there were significant effects of time at the infrapatellar fat pad, at the vastus lateralis, and at the tibialis anterior muscles (*P* < 0.0001; Figure 2).

At the infrapatellar fat pad the PPT significantly increased from test 2 to test 3 (mean difference 41.2 kPa [95% CI 20.2 to 62.2], *P* = 0.0001) and further from test 3 to test 4 (mean difference: 41.7 kPa [95% CI 20.5 to 62.9], *P* = 0.0002, Figure 2).

At the vastus lateralis the PPT significantly increased from test 2 to test 3 (mean difference 37.6 kPa [95% CI 17.9 to 57.4], *P* = 0.0002, Figure 2).

At the tibialis anterior the PPT significantly increased from test 2 to test 3 (mean difference 23.6 kPa [95% CI 0.8 to 46.3], *P* = 0.042) and further from test 3 to test 4 (mean difference: 26.1 kPa [95% CI 3.2 to 49.0], *P* = 0.026, Figure 2).

When excluding the 4 subjects that had fluid aspirated, the results did not change significantly (data not shown).

### 3.2. Manual Pressure Algometry

**Figure 3:**
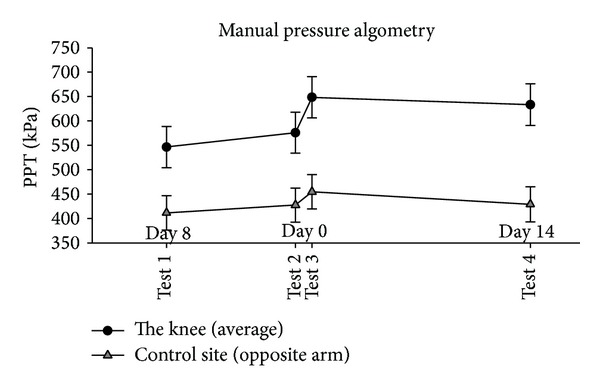
Mean manual PPTs (95% CI, *N* = 25 at tests 1, 2, and 3; *N* = 24 at test 4) at the knee (filled circles) and the contralateral arm (control site—open triangles) before (baseline tests 1 and 2), immediately after (test 3), and two weeks after (test 4) the injection of lidocaine and glucocorticosteroid. PPT was significantly increased at the knee immediately after (test 3) (*P* < 0.0001) and two weeks after (test 4) (*P* = 0.005), but not at the control site (*P* > 0.084), compared with baseline 2 (test 2).

In the PPTs on the knee, there was a significant effect of time (*P* < 0.0001, Figure 3). Compared to test 2, the PPTs significantly increased immediately after the injection at test 3 (mean difference: 72.5 kPa [95% CI 47.94 to 97.06], *P* < 0.0001, Figure 3) and at test 4 two weeks later (mean difference: 57.2 kPa [95% CI 17.93 to 96.48], *P* = 0.005, Figure 3). At the control site (contralateral arm) there was no significant effect of time (*P* = 0.16, Figure 3). Albeit not reaching statistical significance, the mean difference from tests 2 to 3 was 27.2 kPa [95% CI −3.8 to 58.2] (*P* = 0.084, Figure 3). When excluding the 4 subjects that had fluid aspirated, the results did not change significantly (data not shown).

### 3.3. Current Knee Pain Intensity

**Figure 4:**
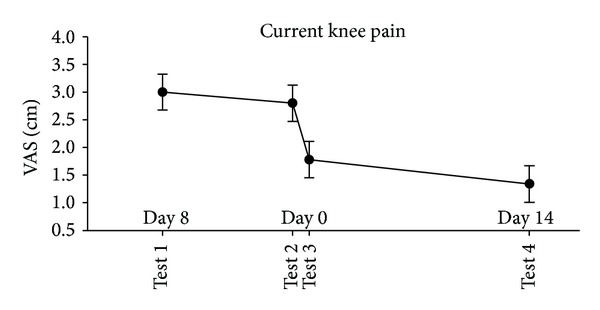
The changes in current knee pain intensity in knee OA patients. Mean VAS scores (95% CI, *N* = 25 at tests 1, 2, and 3; *N* = 24 at test 4) of the pain intensity (VAS scores) measured before (baseline tests 1 and 2) and immediately after the injection of lidocaine and glucocorticosteroid (test 3) and two weeks after the intra-articular injection (test 4).

The VAS scores of current knee pain intensity were significantly reduced immediately after the injection at test 3 and at test 4 two weeks later, compared with tests 1 and 2 (*P* < 0.002, Figure 4). When excluding the 4 subjects that had fluid aspirated, the results did not change significantly (data not shown).

### 3.4. Association of Change in Knee Pain Intensity

The correlations between the manual PPTs at test 2 and the changes in current knee pain VAS scores from test 2 to test 3 were all statistically nonsignificant (the highest correlation coefficient was *r* = 0.09; *P* = 0.65). Similarly, the changes in PPT from test 2 to tests 3 and 4 and changes in current knee pain (VAS) were also not statistically significant (the highest correlation coefficient was *r* = 0.18; *P* = 0.39).

## 4. Discussion

To our knowledge this is the first study to assess the effects of intra-articular analgesia and anti-inflammatory treatment on pain sensitivity in patients with knee OA. There were immediate effects of the intra-articular treatment leading to higher PPTs (reduced pain sensitivity) at the knee and surrounding muscles and this effect was sustained, and in some instances augmented, two weeks after the injection.

The reduced pain sensitivity was not confined to the lower extremity because a trend towards reduced pain sensitivity was observed at the control site on the contralateral arm. There are no definitive models explaining the transition from localized to widespread musculoskeletal pain conditions, but it has been suggested that initial excitation and sensitisation of peripheral nociceptors (e.g., due to joint inflammation) may cause sufficient input to the central pain systems to cause central sensitisation of dorsal horns neurons and/or higher brain centres [15, 16] in line with the present findings showing pressure hyperalgesia in the leg and arm muscles. The regional effects observed in the present study support the notion that input from intra-articular structures may contribute to spreading hyperalgesia. Further, our results may support the proposed involvement of inflammation in the sensitisation phenomena as the effects were sustained after two weeks at both the knee and the surrounding muscles. Widespread hypersensitivity in mechanical pressure pain and loss of pain modulation in patients with symptomatic knee OA were shown to normalise after knee joint replacement [7]. The present study corroborates these findings indicating that simple analgesia and anti-inflammation mimic the effects on the nociceptive system of total knee replacement.

Pain is the principal clinical problem of OA and is generated in the nociceptive system. Studies have documented inflammation in the synovium and other intra-articular structures of OA knees contributing to the sensitisation of pain [17]. Furthermore, inflammation has been shown in periarticular tissue and muscles also contributing to the severity and frequency of OA pain [17, 18]. Although inflammation was not assessed directly, our results suggest that intra-articular anti-inflammatory treatment may have a potential role in preventing spreading hyperalgesia and central sensitisation in patients with knee OA, but this has to be confirmed in controlled trials.

The lack of association between pain sensitivity (PPT), pain sensitivity changes, and improvements in current knee pain could be explained by the difference in the constructs of the two pain assessment types. Clinical pain improvement is influenced by a wide range of parameters, including nociception, psychosocial factors, and affections [19]. Thus, the current daily pain measurement does not necessarily reflect the actual nociceptive mechanisms. In contrast, the PPTs are based on an instant painful stimulation, thus presumably reflecting the nociceptive mechanisms. On the other hand, the sample size may preclude detection of statistically significant associations. This is in contrast with recently reported findings in which higher preoperative widespread pain sensitisation may be associated with chronic pain after total knee replacement [20]; however, our patients were not candidates for surgery. In addition, it is interesting that different results at different sites were obtained with the computer-controlled and manual pressure algometers. The reason might be that the manual algometry is both operator and patient dependent with the inherent variability related to manual pressure application, whereas the computer-controlled is purely patient dependent. Manual algometry is readily available to the broad audience, whereas the computer-controlled algometer is custom made for research purposes only.

Besides the small sample size an important limitation to the present study is the lack of a control group. Thus, placebo effects cannot be ruled out, and placebo might explain the immediate increase in PPT at the control site after injection. Furthermore, it has been shown that intra-articular anesthesia can have analgesic effects for up to 7 days [21], which may confound our findings somewhat. However, these data are the first of their kind and provide important stepping stones for future investigations. The weaknesses of the design were compensated by adding an extra baseline test to assess measurement stability and regression to the mean phenomena before intervening. Intrasession analyses were done to explore a possible significant difference between the two baseline tests in all the analyses, and no significant differences were found (data not shown). Also, the results were robust across multiple methods, assessing pain sensitivity locally and regionally, and with different methods of pressure application (computer-controlled versus manual). This adds strengths to the current findings despite the small sample size and uncontrolled study design.

## 5. Conclusion

Intra-articular analgesia and anti-inflammatory treatment reduced localised and spreading pain sensitivity in patients with knee OA. The effects were immediate and were sustained for at least two weeks. These results should be interpreted with caution for important limitations, such as lack of control group and small sample size.

## Figures and Tables

**Table 1:** Baseline characteristics of the participating patients.

	Mean (SD)	Minimum	Maximum
Age, years	63.4 (8.3)	51	88
Weight, kg	84.2 (15.5)	59.6	116.7
Height, cm	170.9 (8.9)	156.3	191
Body mass index (kg/m^2^)	28.7 (4.3)	20.2	35
Disease duration, years	11.4 (8.0)	1	33
Female, *n* %	20 (80%)		

Data are given as mean, standard deviation (SD), minimum, and maximum unless otherwise indicated.
